# Medicolegal Evaluation of an Abandoned Dead Neonate: A Case Report

**DOI:** 10.31729/jnma.6292

**Published:** 2021-03-31

**Authors:** Alok Atreya, Lokaratna Gyawali, Indira Devkota, Bishnu Pathak

**Affiliations:** 1 Department of Forensic Medicine, Lumbini Medical College, Palpa, Nepal; 2 District Hospital, Palpa, Nepal

**Keywords:** *autopsy*, *crime*, *medicolegal aspects*, *neonaticide*, *Nepal*

## Abstract

**Introduction:** The killing of an illegitimate newborn immediately or within 24 hours of birth is neonaticide which differs from other forms of homicide in terms of diagnosis and motives. Neonaticide is a cognizable offense where mothers are usually the perpetrators. This case reports the autopsy findings of a smothered neonate at a secluded location in rural Nepal. The present case study also highlights the medicolegal implications in such cases.

## INTRODUCTION

The killing of a newborn differs from other forms of homicide in terms of diagnosis and motives. Neonaticide is an act of killing a newborn, immediately or within 24 hours, because the child is not wanted.^1^ The method of neonaticide can be either an act of commission (e.g., strangulation, smothering, poisoning) or an act of omission (e.g., not protecting from heat or cold, not breastfeeding). We present a case of a newborn who was discovered dead in a secluded location in rural Nepal and discuss the medicolegal aspects in such cases.

## CASE REPORT

As per the police inquest, the dead fetus was found near a stream rural hilly region of Nepal at around 6:45 am. The naked body was placed on a brown towellike material above a rock that had reddish stains at multiple places. A red-stained sanitary pad-like material and a sleeveless woolen sweater with floral designs were recovered near the crime scene.

**Figure 1.**
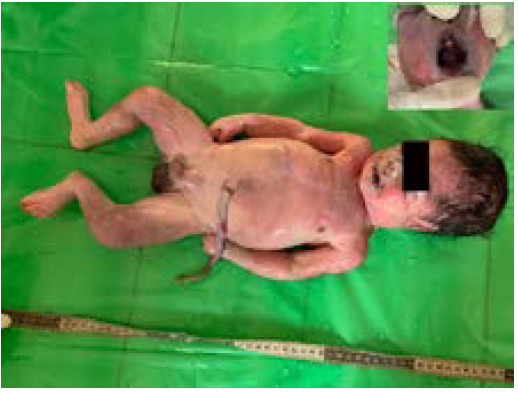
Frontal view of the neonate and lacerated upper jaw (inset).

At autopsy, it was a male baby wrapped in a polythene bag which measured 51 cm in length and weighed 2.9 kg. Post-mortem ant bite created artifacts in form of abrasions at multiple places. There were green meconium stains over the perineal region and lower limbs. The head was diffusely covered with black hair about 1 cm long. The placenta was not recovered. The umbilical cord was severed at 14.5 cm, the severed end of which was dry and had an irregular margin with a hemorrhagic spot at 6 cm from the fetal end. The cheeks were pinkish at the sides; however, the medial part of the face was discolored blue. The lips were dark brown. Brownish discoloration was noted in the oral cavity and nasal orifices. The frenulum and gum margin was seen to be lacerated at the upper jaw (Figure 1).

**Figure 2A, 2B, 2C, 2D.**
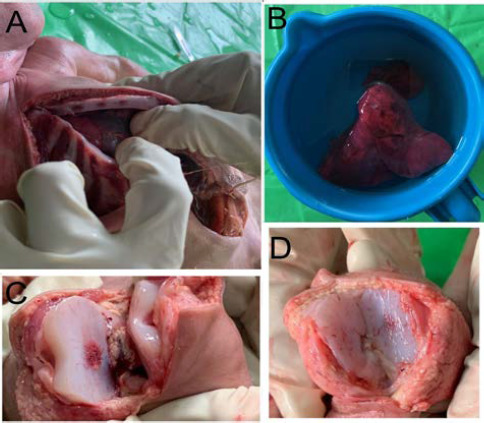
Ossification centers of the body of the sternum and overlapping lung margins (2A), Hydrostatic Test showing the floated lung (2B), Ossification center in distal epiphysis of the right femur (2C), Ossification center in the proximal epiphysis of right tibia (2D).

The neck showed crescentic abrasions at the angle of the jaw on both sides. Post-mortem lividity was evident over the chest, upper abdomen, and anterior aspect of both the lower limbs. Testicles were descended in the scrotum. Fingernails extended beyond the fingertips. No gross congenital anomaly was noted. On opening the abdomen, the highest point of the diaphragm was noted between the fifth and sixth rib space. The lungs were light brown in color with the medial round margins overlapping each other anteriorly (Figure 2A). They were spongy in consistency. When the lungs were immersed in water they floated, giving a positive hydrostatic test (Figure 2B). The stomach contained mucus without any abnormal odor. Ossification centers for the body of sternum (Figure 2A), calcaneum, distal epiphysis of the femur (Figure 2C), and proximal epiphysis of the tibia were present (Figure 2D). The rest of the findings were unremarkable. Samples were preserved and handed over to the investigating officer for DNA analysis.

## DISCUSSION

During the autopsy of a newborn, forensic pathologists are required to determine if it was a case of live birth, stillbirth, or dead born. In the present case the biometric measurements, presence of ossification centers, and the fact that testicles were descended positively point that the fetus was full-term when delivered. There were no signs of maceration which ruled out the case to be of intrauterine death.

There was no malformation noted which could have made intrauterine life incompatible. The expanded lungs with round margins, the low-lying diaphragm, and positive ‘hydrostatic test’ were indicative of respiration.

The umbilical cord also gives a clue about the circumstances of birth. The cord is clean-cut and clamped in case of hospital delivery. When the mother is unaware or not prepared for the delivery, she would not have a sharp cutting instrument to cut the umbilical cord. Deliberate attempts at tearing the cord with bare hands or pinching with nails to breakthrough will make the end margins of the umbilical cord appear irregular.

From the legal perspective, a fine distinction is required to prove or preclude live birth because legal prosecution will vary in both circumstances. Aeration of the lungs is considered a surrogate marker of live birth. It is argued that air can enter the lungs after the rupture of the amniotic membrane before the child is completely born.^2^

Furthermore, resuscitation attempts at birth can also cause air entry into the lungs. There are fallacies of hydrostatic tests that have come negative in cases of live birth.^2,3^ Laceration of the umbilical cord too isn't a concrete finding in a case of neonaticide. In the case of precipitate labor, when the delivery is without mother's knowledge as all the stages of labor merge into one, sudden delivery may snap the umbilical cord leaving behind the lacerated margin. Complete delivery of the fetus and placenta however is a common finding in cases of precipitate labor.^4,5^

Mothers are the offenders in almost all cases of neonaticide.^1^ The rupture of the frenulum, congested face, and meconium discharge point towards the smothering of the newborn in the present case. The act of neonaticide by smothering in the present case is likely by the mother who had delivered the child based upon the circumstantial evidence of recent delivery at a secluded location and other corroborative evidence of live birth. The DNA samples obtained from the fetus during the autopsy will help in the positive identification of the mother.

In the present case, it could not be established the ulterior motive behind neonaticide. A review of forty years on infanticide and neonaticide revealed that mothers who have killed their newborn do so either to get rid of the unwanted child or to take revenge from the child's father.^6^ Mental illness of mother is also attributed to cases of infanticide. However, it has been observed that mothers who wanted to have a free life unencumbered by an infant, did end up killing their child.^6,7^

The patriarchal societal nature and the stigma of pregnancy in an unmarried, widowed, or extramarital relationship forces the mother to ruthless killing and disposal of her child. The legal system in Nepal too punishes the women for neonaticide, however, the offending male who made her pregnant will never be interrogated or punished.^8^

As per the Nepalese Muluki Criminal Code, Chapter on Homicide, Article number 184(1), one should not abandon or leave behind a newborn.^9^ If found guilty, the offender can be slammed with three years of imprisonment and a fine of thirty thousand rupees. As per article 184(2) of the same section, if the abandoned newborn dies then the offender will be prosecuted as per the law on homicide as stated in Article 177 and punished with life imprisonment.
